# Clinical significance and biological function of PRKCQ-AS1/miR-582-3p expression in LUAD

**DOI:** 10.1186/s41065-025-00482-9

**Published:** 2025-07-01

**Authors:** Lingling Liu, Xiaofen Liu, Xiaojiao Wu, Hang Fang, Jingjing Shi, Wei Jiang

**Affiliations:** 1 Integrated Chinese and Western Medicine Oncology, the First Hospital of Qiqihar, Qiqihar, 161000 China; 2 Department of Respiratory and Critical Care Medicine, Taikang Tongji (Wuhan) Hospital, Wuhan, 430050 China; 3 Department of Laboratory, The First People’s Hospital of Yongkang, Yongkang, 321300 China; 4 Department of Respiratory Medicine, Yantai Yuhuangding Hospital, No. 20, Yuhuangding East Road, Zhifu District, Yantai City, 264000 Shandong Province China

**Keywords:** PRKCQ-AS1, LUAD, miR-582-3p

## Abstract

**Objective:**

To investigate the clinical value and mechanism of action of long non-coding RNA PRKCQ-AS1 for lung adenocarcinoma (LUAD) progression.

**Methods:**

Clinical data of 128 LUAD patients were collected, postoperative pathological tissues were stored at -80 °C. Kaplan-Meier survival analysis was employed to investigate differences in 5-year survival rates across various expression groups, while Cox regression models assessed the prognostic factors influencing patient outcomes. Reverse transcription quantitative PCR (RT-qPCR) was utilized to measure the expression levels of PRKCQ-AS1 and miR-582-3p in pathological tissues and LUAD cell lines. Additionally, a dual-luciferase reporter assay validated the reciprocal relationship. CCK8 examined cell proliferation, Transwell observed cell migration and invasion.

**Results:**

PRKCQ-AS1 was down-regulated and miR-582-3p was up-regulated in LUAD tissues and cell. PRKCQ-AS1 and miR-582-3p expression affects some pathological features (lymph node metastasis, TNM stage, tumour differentiation) in LUAD patients. Patients with low PRKCQ-AS1 and high miR-582-3p had increased mortality. Interaction of PRKCQ-AS1 targeting miR-582-3p exists in LUAD cells. RGMB, STXBP6 are downstream target genes of miR-582-3p. Overexpression of (oe-) PRKCQ-AS1 inhibited LUAD cell proliferation, migration, and invasion. However, concomitant use of miR-582-3p mimics resisted the effects of PRKCQ-AS1 overexpression on cells.

**Conclusion:**

PRKCQ-AS1/miR-582-3p axis regulatory relationship exists in lung adenocarcinoma cells. PRKCQ-AS1 may regulate the proliferation, migration and invasion of lung adenocarcinoma cells and participate in LUAD regulation by targeting miR-582-3p.

**Clinical trial number:**

Not applicable.

**Supplementary Information:**

The online version contains supplementary material available at 10.1186/s41065-025-00482-9.

## Introduction

Lung adenocarcinoma (LUAD) is a prevalent subtype of non-small cell lung carcinoma and represents a significant challenge within clinical oncology [1]. LUAD occurs mostly in the smoking population, and due to the rapid progression and aggressiveness of the disease, the overall survival (OS) of patients with advanced stage is generally no more than 5 years [2, 3]. In clinical practice, medication and conservative treatment are usually used to stop patients from deteriorating [4], but this approach is only effective in treating patients with early-stage LUAD. When LUAD progresses more seriously, radiotherapy or chemotherapy is often used [5]. However, radiotherapy has strong side effects such as nausea, dizziness, hair loss, etc. [6], especially for already weak cancer patients. Given the current landscape of LUAD management, where no definitive cure exists, the urgent need to identify new therapeutic markers and explore innovative targets for treatment is paramount. It is within this context that the search for effective interventions gains critical importance, heralding a new era of potential advancements in the fight against this formidable malignancy.

In recent years, the significance of non-coding RNAs (ncRNAs) has garnered increasing attention as their functions become more comprehensively understood [7–9]. Despite their lack of direct involvement in the synthesis of pivotal proteins within organisms, their crucial roles in various biological activities are gradually being elucidated. It can participate in cellular growth and development, modulate tissue metabolism [10], and regulate tumor progression alongside the onset of various diseases [11]. For instance, lncRNA LINC02323 demonstrates elevated expression levels in gastric cancer cells, correlating with poor prognosis outcomes and malignant progression in affected patients [11]. Similarly, mALAT1 silencing induces aberrant proliferation in multiple myeloma (MM) by inhibiting CXCR4/CXCL12 [12]. In the context of pulmonary disease, chronic non-obstructive pulmonary disease (COPD) exhibits a notable propensity to progress into LUAD [13]. An analysis of co-expression RNA networks in COPD patients showed that PRKCQ-AS1 is abnormally expressed and may play a critical role in the progression of this disease [14]. In our exploration of PRKCQ-AS1, we uncovered its involvement in various other diseases as well. For example, PRKCQ-AS1 targets miR-1287-5p/YBX1 to promote the proliferation of colorectal cancer cells [15]. Additionally, it influences the prognosis of sepsis patients through its regulatory effects on immune processes [16]. However, we found no existing information detailing the regulatory role of PRKCQ-AS1 in LUAD.

MicroRNAs (miRNAs) have frequently been reported as key players in lung cancer progression [17]. For example, miR-582-3p effectively inhibited the malignant transformation of non-small cell carcinoma by up-regulating the expression of the downstream target gene RGMB [18]. Moreover, the repression of miR-582-3p consistently diminishes the proliferative capacity of lung cancer cells and induced apoptosis [19]. RNA protein tyrosine phosphatase receptor induces apoptosis in LUAD cells via miR-582-3p [19]. Furthermore, preliminary laboratory analyses utilizing the StarBase database have revealed that the sequence of PRKCQ-AS1 possesses multiple binding sites that with the miR-582-3p sequence. Therefore, we guess that PRKCQ-AS1 may target miR-582-3p involved in the disease progression of LUAD. Therefore, it is necessary to collect relevant cases to further explore the diagnostic and regulatory mechanisms of PRKCQ-AS1 for LUAD.

Based on the above background, this study analyzed the prognostic value of PRKCQ-AS1 for LUAD by collecting clinical information on LUAD cases. The downstream target genes of PRKCQ-AS1 and miR-582-3p were predicted by StarBase prediction and TargetScan database, respectively. Four different lung cancer cells were used to investigate in depth the regulatory role and biological function of PRKCQ-AS1 in LUAD disease progression.

## Materials and methods

### Study subjects

Lung adenocarcinoma and associated paraneoplastic tissues were meticulously collected from 128 patients who underwent surgical treatment at The First People’s Hospital of Yongkang between February 2017 to January 2018. The sample size required for the experiment was calculated using GPOWER statistical software, with a Type I error of 0.05 and a beta error of 0.2. The sample size was calculated to be 103, with 80% power. These specimens were subsequently preserved at a temperature of -80℃. The patients’ prognosis and mortality were diligently monitored for a span of 5 consecutive years. The study was approved by the Ethics Committee of The First People’s Hospital of Yongkang. During the course of the study, we meticulously documented the patients’ basic information and relevant medical histories ensuring that all sensitive information remained confidential. All patients were graded for inclusion according to the results of pathological sections, and none of them were treated with drugs or radiation before surgery. Participating patients gave informed consent and signed a written consent form in accordance with the Declaration of Helsinki. The inclusion criteria for the study were (1) Adult patients (non-pregnant) aged 18–75 years. (2) Complete medical data and follow-up records. (3) No history of drug and surgical treatment. Patients with mixed adenocarcinoma, patients with metastatic malignancy, patients with coagulation disorders, cardiopulmonary insufficiency, and patients with psychiatric disorders were excluded from the study.

### Real-time fluorescence quantitative PCR(RT-qPCR)

Cancer tissues, stored in a -80 °C refrigerator, were meticulously homogenized on ice using a Tissue Master™ High-Throughput Tissue Mill (E6681, Beyotime, Shanghai, China). Following this, the tissue samples were supplemented with Trizol reagent (Invitrogen, USA) adhering to specified guidelines based on the gram weight or the number of cells collected. Total RNA was extracted according to the manufacturer’s instructions, with RNA concentration and purity assessed using an Ultra-Micro UV Spectrophotometer (MicrospectroMD-1000). Total cDNA was synthesized using 2 µg of RNA as a template according to the instructions of Hifair^®^ Advance Fast 1st Strand cDNA Synthesis Kit (11150, Next Sense Biologicals) under the following conditions: 55 °C for 5 min, 85 °C for 5 s. One Step Prime Script II Fluorescence Quantification Kit (RR600A, TaKaRa, Japan) was used to perform qPCR reaction under the following conditions: 52 °C, 5 min, 95 °C, 10s preheating, 95 °C, 5s, 60 °C, 30s, with a total of 40 cycles, and the 7500 Fast Real-Time Fluorescence PCR System (Thermo, USA) to detect the expression of the relevant genes. The primer sequence for PRKCQ-AS1 (NR_036501) was designed as forward: 5’-ACTGCTTTCAACTTTACTG-3’, reverse: 5’- AGTCCTCAGCATTATTCC-3’, and the primer sequence corresponding to the internal reference GAPDH was according to previous studies [16]: forward: 5’-CATTTCCTGGTATGACAACGA-3’, reverse: 5’- GGGTCTTACTCCTTGGAGG-3’. In addition, primers for miR-582-3p and its internal reference U6 were purchased from Ribobio [20] (miR-582-3p: MQPS0001915-1-100; U6: miRAN0002-1-100).

### Cell culture and transfection

The experimental study utilized five distinct of cell lines, including four types of human LUAD cells (PC-9, A549, H-2009, H-1299) and human bronchial epithelial cells HBE, all procured from Sunn Bio. Specifically, PC-9, H-1299 and HBE cells were cultured in a medium consisting of 1640 supplemented with 10% fetal bovine serum (FBS) and 1% penicillin/streptomycin (P/S). A549 cells were maintained in DMEM + 10% FBS + 1% P/S, while H-2009 cells were cultivated in a DMEM/F12 (1:1) + 5% FBS + 1% P/S in addition to 5 µg/ml insulin, 10 nM hydrocortisone, 10 nM β-estradiol. All cell lines were cultured at 37 °C under a humidified atmosphere of 95% O_2_, 5% CO_2_.

In preparation for the experiment, two types of lung adenocarcinoma (LUAD) cells, PC-9 and A549, were inoculated into 24-well plates. Upon reaching a confluence of approximately 70%, the cells were transfected using Lipofectamine™ 3000 (Lipo, Invitrogen, USA). To initiate the transfection process, Lipo 3000 was diluted with serum-free medium within tube A of the premix. Meanwhile, the oe-PRKCQ-AS1 and oe-miR-mimic were diluted in serum-free medium and P3000 in tube B. An equal volume of the premix in tube B was poured into tube A and incubated at room temperature for 10 min. The cells were then stimulated with 500 ng of DNA per well from the premix. Following this, the 24-well plate was incubated in a constant temperature cell culture incubator for 6 h. The experiment could be continued after 24 h of growth by changing the fresh medium.

### Dual luciferase reporter assay

The StarBase database (https://rnasysu.com/encori/) prediction results showed that miR-582-3p is a downstream target gene of PRKCQ-AS1, the TargetScan database (http://www.targetscan.org) predicted RGMB, STXBP6 as target genes of miR-582-3p, but whether there is a reciprocal relationship between the two needs to be further verified. Firstly, in this experiment, a wild-type PRKCQ-AS1 (WT- PRKCQ-AS1,5’ -ACAGCUUGGAGGAAACCAGUUUU-3’) plasmid was constructed by pGL3-Basic Vector with mutant PRKCQ-AS1 (MT- PRKCQ-AS1, 5’-ACAGGUUGGAGGAUUGGUCAAU-3’). WT/MUT-RGMB and WT/MUT-STXBP6 plasmids were constructed by pmirGLO luciferase vector (Promega, Madison, USA). The reporter plasmid, together with an internal reference gene, was co-transfected into LUAD cells using Lipo 3000, followed by incubation under appropriate conditions for 48 h. Subsequently, the cells were lysed, and 20 µl of the supernatant was transferred to a 96-well luminometer plate protected from light. The relative fluorescence intensity of the supernatant was measured using a multifunctional enzyme marker after the addition of the corresponding fluorescein detection reagent. Normalization of the experimental results was conducted based on the luminescence intensity of the reference signal from the sea kidney.

### Cell proliferation assay

Cell proliferation assay was detected by Cell Counting Kit-8 (C0038, Beyotime, Shanghai, China). Cells were initially seeded in 96-well plates at a density of approximately 5,000 cells per well in 100 µl of cell suspension, and were subsequently placed in a constant temperature incubator for 24 h to allow for cell adherence to the plate surface. The oe-NC, oe-PRKCQ-AS1, miR-NC, and miR-mimic were transfected into two kinds of LUAD cells by liposomes, and incubated in the incubator for 12 h. After the transfection period, 10 µl of CCK8 reagent was added to each well, followed by gentle shaking to ensure thorough mixing. The cells were incubated for 2 h in a thermostatic incubator with 95% O_2_, 5% CO_2_. The proliferation of different groups of cells was measured at 450 nm absorbance using a Multiskan Sky High full-wavelength enzyme labeler.

### Transwell test for cell migration and invasion

Two types of LUAD cells, transfected with distinct plasmids and cultivated to a confluence of 70% or greater in conventional conditions, were harvested and resuspended using a serum-free medium. Subsequently, the cells were inoculated in the upper chamber of a transwell at a density of 1 × 10^5^/well. In the lower chamber, 200 µl of basal medium enriched with 20% serum was introduced. The plates were then incubated in a controlled atmosphere of 95% O_2_ and 5% CO_2_ at 37 °C for a duration of 4 h. The cells were washed with PBS and stained at the endpoint, and the polycarbonate membranes in the chambers were fixed in a 4% paraformaldehyde(PFA)solution for 5 min. The cell migration was observed under an inverted microscope using a neutral dendrimer preparation.

The cells were cultured in serum-free medium for a duration of 24 h prior to the invasion assay. Concurrently, a matrix gel was applied to the bottom of the upper chamber of the small wells and incubated in an incubator for 3 h to allow for proper adhesion. Following this, 100 µl of serum-free medium was added to the upper chamber and incubated for an additional 30 min to facilitate the hydration of the basement membrane. Cells, resuspended in serum-free medium, were then inoculated into the upper chambers of the wells at a consistent density. Meanwhile, the lower chambers received 500 µl of complete medium supplemented wat a consistent density 10% serum and were incubated for 24 h. Liquid in the chamber was discarded and washed with PBS for fixation with paraformaldehyde, and the bottom of the chamber was immersed in 0.1% crystal violet staining for 5 min, washed in pure water wiped by cotton swabs, and placed under the microscope with a small number of droplets. The number of cells in 5 fields of view was recorded on the monitor after PBS, and the average value was taken.

### RNA immunoprecipitation test (RIP)

The interactions between PRKCQ-AS1 and miR-582-3p were verified by RNA Immunoprecipitation (RIP) kit (Absin, Shanghai, China). PBS was used to wash the adherent cells, trypsin digestion was performed, and the precipitate was collected by centrifugation. Discard the supernatant, add Complete RIP Lysis Buffer (protease inhibitor and RNAase inhibitor have been added) to the remaining precipitate, vortex the cells vigorously in an ice bath to rest, and repeat several times. centrifuge the cells at 4 °C, 12,000 × g for 8 min, and aspirate the supernatant in a new centrifuge tube. Add 4 µl of target antibody (anti-PRKCQ-AS1/miR-582-3p) to the IP tube containing magnetic beads and add 4 µl of host isotype antibody IgG to the control tube. close the mouth of the tube and incubate on a rotary mixer for 2 h. After standing, gently remove the supernatant and add 300 µl of washing buffer to the tube and repeat the process several times to remove unbound material and non-specific impurities. Repeat the process several times to remove unbound material and non-specific impurities. Add 100 µl Elution Buffer to each tube to elute the target protein-RNA complex captured by the magnetic beads. Total RNA in the eluate was extracted by TRIzol and detected by RT-qPCR.

### Data analysis

SPSS and GraphPad Prism 9 were used to process and analyze the data information. All statistical evaluations comprised three biological replicates accompanied by technical replicates, and the results were expressed as mean ± standard deviation (x ® ± s). To compare samples between groups, a T-test was employed. Kaplan-Meier was utilized to assess the difference in survival over 5 years between the two groups of patients with high and low expression, while Cox regression analyses were performed to identify prognostic factors affecting patient outcomes. Fluorescence real-time quantitative results were analyzed by 2^−ΔΔCt^ for relative quantification. Significant differences in the statistical results were defined as *P* < 0.05.

## Results

### Relationship between PRKCQ-AS1 and miR-582-3p and pathological features of LUAD patients

Based on the above background, this present study analyzed the relationship between PRKCQ-AS1 and miR-582-3p expression and the clinicopathological characteristics of LUAD patients. Among them, lymph node tissue samples were obtained through surgical resection, and the presence of cancer cells in the lymph nodes was directly observed to determine lymph node metastasis. According to the standards of the International Union Against Cancer (UICC) [21], the size, location, number, and extent of the tumor were divided into TNM stages, and the patients were classified into I-IV. The degree of tumor differentiation was observed based on pathological sections of surgically obtained lung adenocarcinoma samples. Tumors with morphology and structure close to normal cells were considered highly differentiated, and those with large differences from normal cells were considered poorly differentiated. The statistical results of 128 cases showed that there was no significant difference between the changes in PRKCQ-AS1 and miR-582-3p expression and the gender, age, smoking history, and tumor size of LUAD patients. However, a noteworthy observation emerged regarding the incidence of lymph node metastasis, TNM stage, and neoplasm differentiation in LUAD patients, wherein those classified within the PRKCQ-AS1 low-expression group (Table 1) and the miR-582-3p high-expression group (Table 2) exhibited more pronounced severity than their counterparts in the other groups (*P < 0.05*).

**Table 1:** The association of PRKCQ-AS1 with patients’ clinicopathological features

Variant	Cases (*n* = 128)	PRKCQ-AS1 expression		*P*
		Low (*n* = 66)	High (*n* = 62)	
**Gender**				0.093
Male	75	34	41	
Female	53	32	21	
**Age**				0.982
< 60	60	31	29	
≥ 60	68	35	33	
**Smoking history**				0.199
No	57	33	24	
Yes	71	33	38	
**Tumour size(cm)**				0.457
≤ 3	70	34	36	
> 3	58	32	26	
**Differentiation**				**0.032**
Well-moderate	83	37	46	
Poor	45	29	16	
**Lymph node metastasis**				**0.024**
No	89	40	49	
Yes	39	26	13	
**TNM stage**				**0.026**
I-II	87	39	48	
III	41	27	14	
Neoplasm stage				0.300
T1-T2	83	40	43	
T3-T4	45	26	19	

*P* < 0.05 indicates a statistically significant difference in the data

**Table 2:** The association of miR-582-3p with patients’ clinicopathological features *P* < 0.05 indicates a statistically significant difference in the data

Variant	Cases (*n* = 128)	miR-582-3p expression		*P*
		low (*n* = 65)	High (*n* = 63)	
**Gender**				0.078
Male	75	43	32	
Female	53	22	31	
**Age**				0.587
< 60	60	32	28	
≥ 60	68	33	35	
**Smoking history**				0.295
No	57	26	31	
Yes	71	39	32	
**Tumor size(cm)**				0.606
≤ 3	70	37	33	
> 3	58	28	30	
**Differentiation**				**0.011**
Well-moderate	83	49	34	
Poor	45	16	29	
**Lymph node metastasis**				**0.026**
No	89	51	38	
Yes	39	14	25	
**TNM stage**				**0.027**
I-II	87	50	37	
III	41	15	26	
Neoplasm stage				0.493
T1-T2	83	44	39	
T3-T4	45	21	24	

### Effect of PRKCQ-AS1 and miR-582-3p expression on prognosis and survival of LUAD patients

**Fig. 1:**
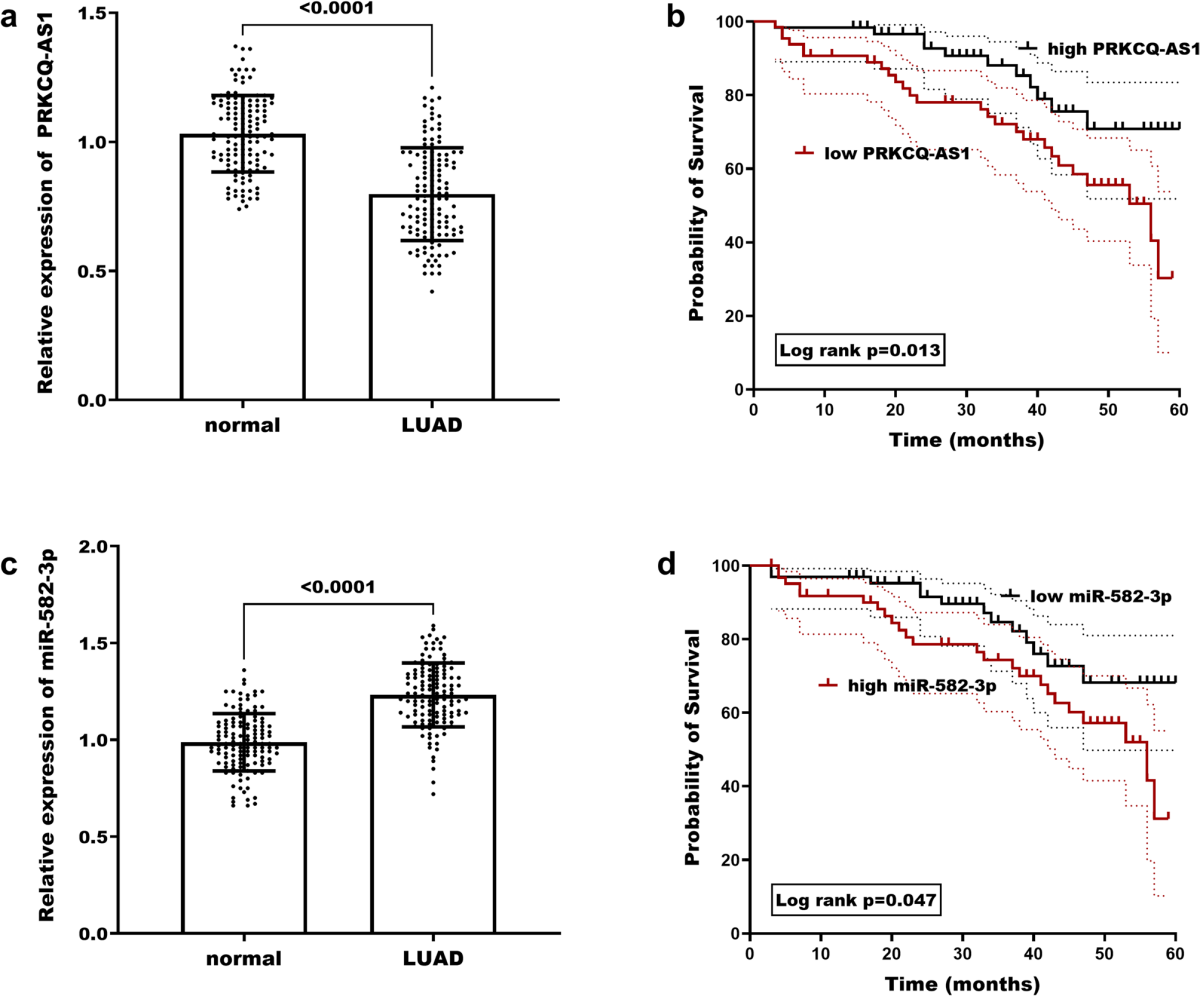
Illustrates the impact of PRKCQ-AS1 and miR-582-3p expression on lung cancer patients and their survival outcomes. (**a**) Displays the variations in PRKCQ-AS1 expression in normal tissues and LUAD. (**b**) Examines the correlation between differing levels of PRKCQ-AS1 expression and survival outcomes for LUAD patients. (**c**) Highlights the alterations in miR-582-3p expression observed in normal tissues compared to LUAD. (d) Investigates the relationship between variable expression levels of miR-582-3p and the survival rates of LUAD patients. *P* < 0.05 indicates a statistically significant difference in the data

Quantitative analysis was performed on frozen tissues from normal subjects and LUAD patients. The expression of PRKCQ-AS1 was found to be markedly decreased in LUAD patients (Figure. 1a, *P* < 0.001), whereas miR-582-3p exhibited a significant upregulation in cancerous tissues compared to normal tissues (Figure. 1c, *P* < 0.0001). Kaplan-Meier log-rank tests showed that LUAD patients with reduced levels of PRKCQ-AS1 expression and elevated miR-582-3p expression exhibited poorer survival outcomes (*P < 0.05*, Fig. 1b and d). Furthermore, multivariate Cox regression analysis identified differentiation, lymph node metastasis, TNM stage, along with PRKCQ-AS1 (*P < 0.01*), and miR-582-3p (*P < 0.05*) as significant risk factors affecting the prognostic profile of LUAD patients (Table 3).

**Table 3:** Analysis of factors affecting the cumulative survival of LUAD

Characteristics	Multivariate Cox’s regression			
	*P* value	HR	95%	
**PRKCQ-AS1**	**0.001**	0.042	0.007–0.260	
**miR-582-3p**	**0.013**	8.7	1.575–48.04	
Gender	0.312	1.449	0.706–2.972	
Age	0.590	1.217	0.595–2.489	
Smoking history	0.319	1.428	0.709–2.875	
Tumor size(cm)	0.685	1.157	0.572–2.341	
**Differentiation**	**0.035**	2.476	1.067–5.748	
**Lymph node metastasis**	**0.030**	2.32	1.086–4.956	
**TNM stage**	**0.029**	2.603	1.104–6.138	
Neoplasm stage	0.195	1.638	0.776–3.458	

*P* < 0.05 indicates a statistically significant difference in the data

### Expression of PRKCQ-AS1 and miR-582-3p in LUAD cells

**Fig. 2:**
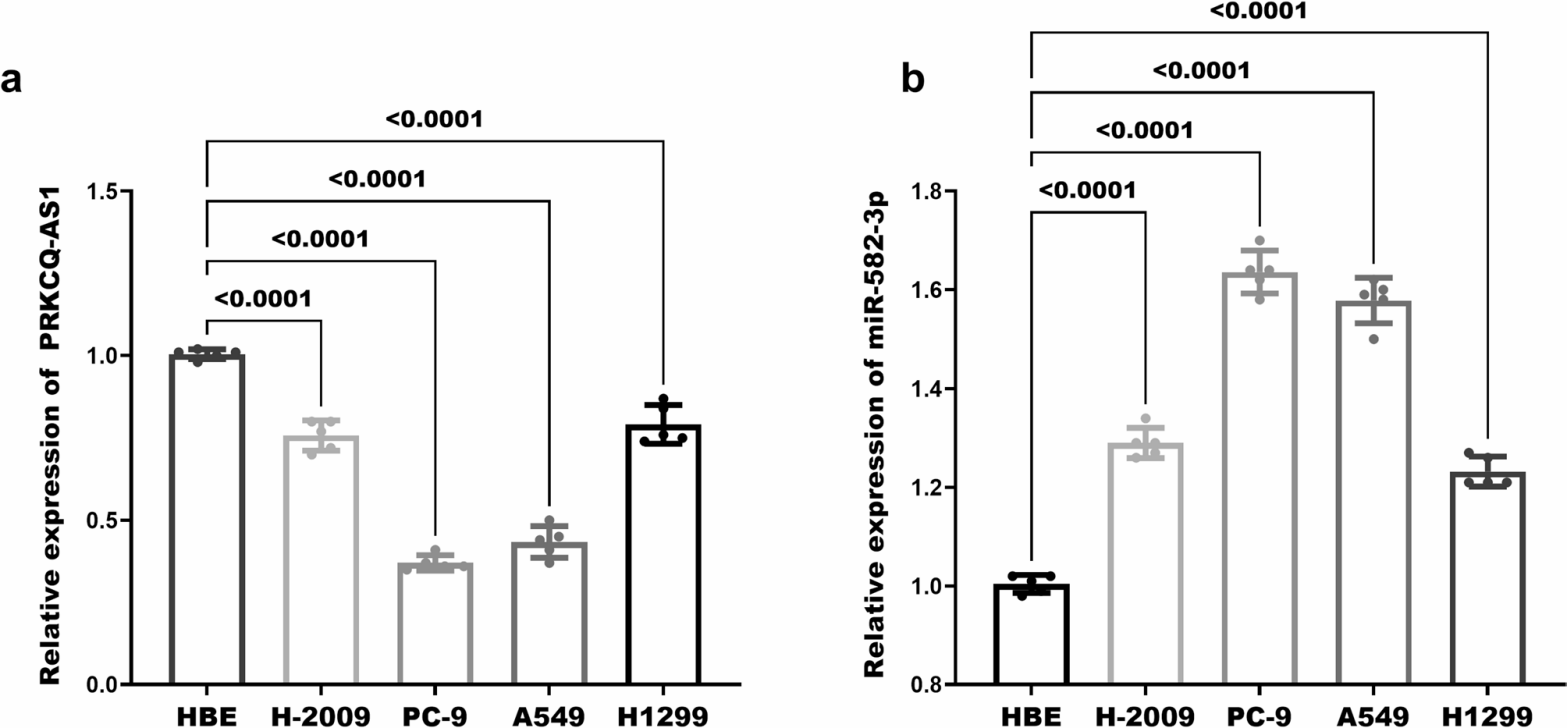
Illustration of the expression levels of PRKCQ-AS1 with miR-582-3p in lung cells. (**a**) Comparative expression of PRKCQ-AS1 between normal lung cells and LUAD cells, (**b**) Variations in the expression of miR-582-3p in normal lung cells versus LUAD cells. Where HBE are normal human bronchial epithelial cells and H-2009, PC-9, A549, and H1299 are LUAD cells. *P* < 0.05 indicates a statistically significant difference in data

In order to verify the above clinical results, we verified the expression changes of PRKCQ-AS1 and miR-582-3p in LUAD cell lines, namely H2009, PC-9, A549, H1299, alongside normal bronchial epithelial cells (HBE) adjacent to the carcinoma. The results obtained from RT-qPCR revealed a significantly reduced expression of PRKCQ-AS1 in the four LUAD cell lines when compared to the HBE cells (*P < 0.0001*, Fig. 2a). Conversely, the expression of miR-582-3p was markedly higher in LUAD cells relative to HBE cells (*P < 0.001*, Fig. 2b). This result further demonstrated the existence of PRKCQ-AS1 low expression with miR-582-3p upregulation in LUAD.

### Relationship between PRKCQ-AS1 and miR-582-3p in LUAD

**Fig. 3:**
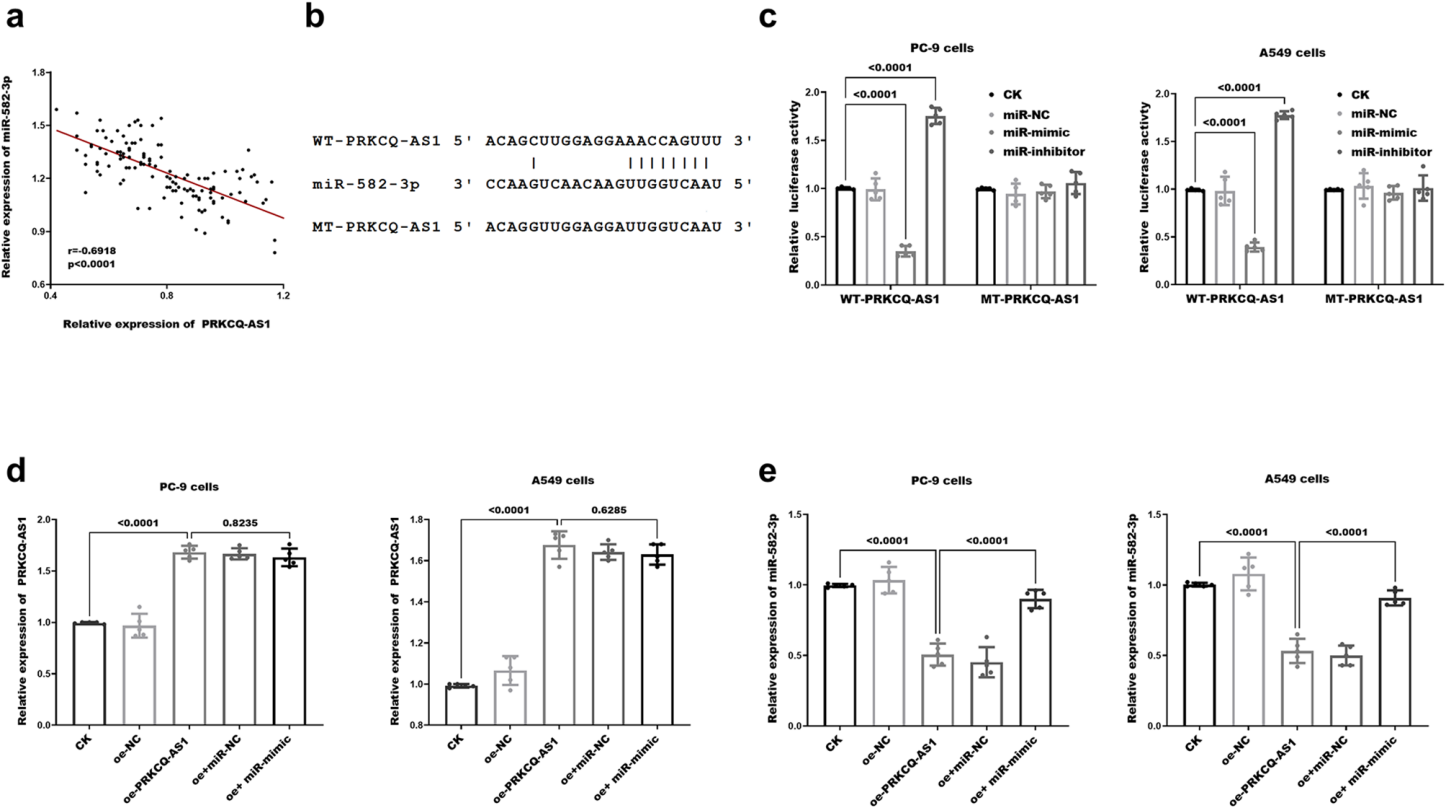
Validation of the interactions between PRKCQ-AS1 and miR-582-3p. (**a**) Pearson correlation analysis was conducted to assess the relationship between the expression levels of PRKCQ-AS1 and miR-582-3p in tumor tissues of LUAD. (**b**) Sequence construction of wild-type (WT) and mutant (MT) PRKCQ-AS1 vectors. (**c**) Fluorescence intensity variations of two LUAD cell types harboring WT- versus MT-PRKCQ-AS1 were evaluated following transfection with miR-NC, miR-mimic, or miR-inhibitor. Expression levels of PRKCQ-AS1 (**d**) and miR-582-3p (**e**) in both LUAD cells types were measured post-transfection with oe- PRKCQ-AS1, oe + miR-mimic. Where oe- denotes the overexpression vector of the gene, -mimic denotes the mimic with the same effect as miRNA, and *P* < 0.05 denotes a statistically significant difference in the data

Correlation analysis demonstrated a significant inverse relationship between PRKCQ-AS1 and miR-582-3p levels in patients with tumor tissues (*r* = -0.6918, *P* < 0.0001, Fig. 3a). To identify potential downstream target miRNAs of PRKCQ-AS1, the StarBase database (https://rnasysu.com/encori/) was employed, and the corresponding binding site is illustrated in Fig. 3b. Dual luciferase reporter assay showed that the fluorescence activity in LUAD cells containing wild-type PRKCQ-AS1 significantly decreased following transfection with miR-mimic, while it markedly increased upon the introduction of miR-inhibitor (Fig. 3c). No significant changes in fluorescence values were observed in LUAD cells harboring mutant PRKCQ-AS1. Additionally, PRKCQ-AS1 and miR-582-3p were enriched in the Ago2 group compared to the IgG group (*P<0.001,* Supplement Figure 1). In addition, the expression of PRKCQ-AS1 was increased and miR-582-3p levels was suppressed in LUAD cells transfected with oe-PRKCQ-AS1. Notably, the repressed miR-582-3p expression could be notably restored by co-transfecting with miR-mimic (*P < 0.0001*, Fig. 3d-e). This result suggests that miR-582-3p can directly interact with PRKCQ-AS1 in LUAD.

### Effect of PRKCQ-AS1/miR-582-3p interaction on LUAD cell function and prediction of miR-582-3p targets

**Fig. 4:**
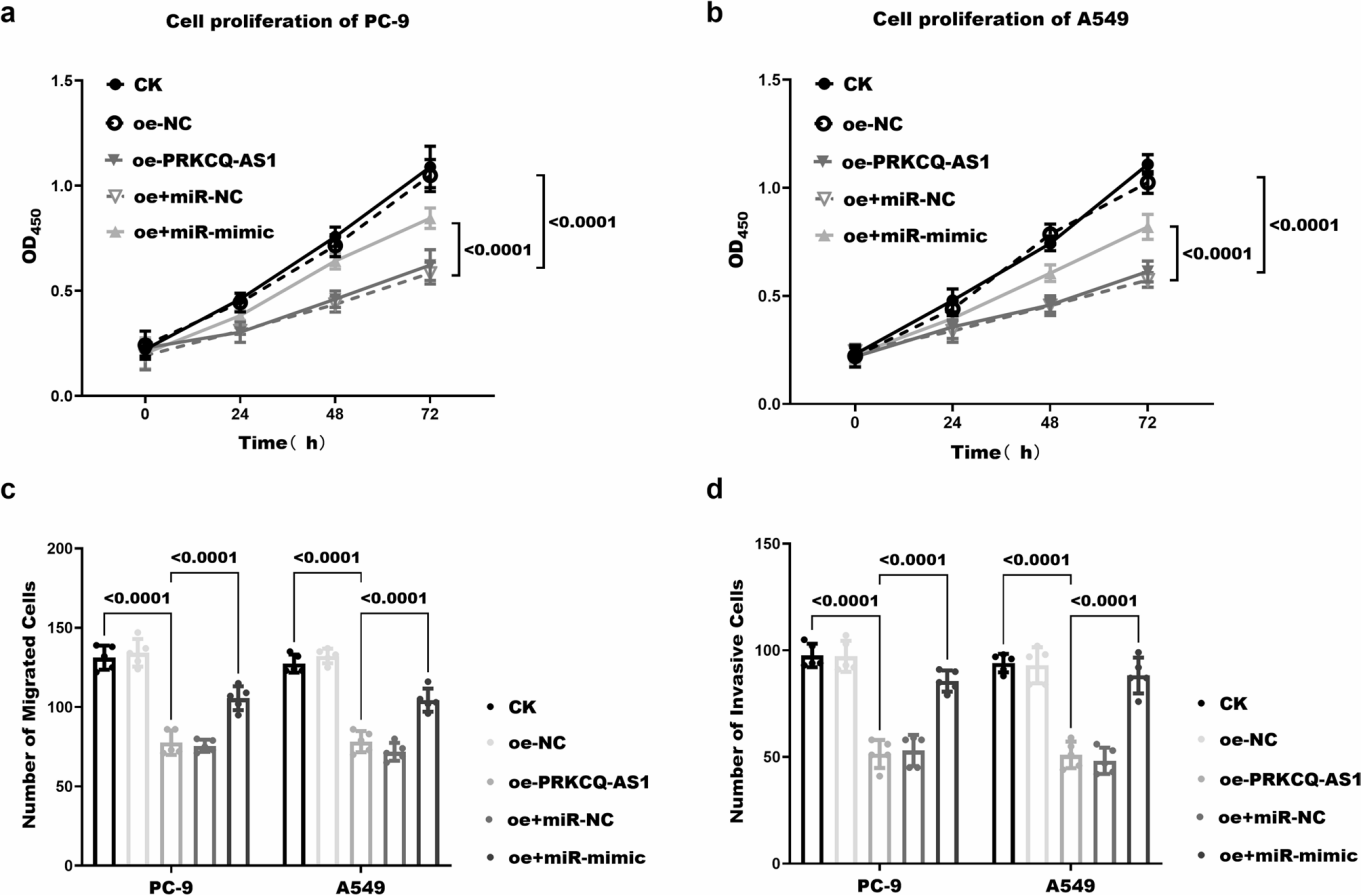
Functional study of PRKCQ-AS1/miR-582-3p regulation in LUAD cells. The proliferative capacity of PC-9 (**a**) and A549 (**b**) cells was assessed following transfection with oe- PRKCQ-AS1, coupled with the concurrent application of miR-mimic, measured at OD = 450 nm. Additionally, migration capacity (**c**) and invasive capacity (**d**) of LUAD cells were evaluated post-overexpression of PRKCQ-AS1 and alongside the administration of miR-582-3p mimic. *P* < 0.05 indicates a statistically significant difference in data

**Fig. 5:**
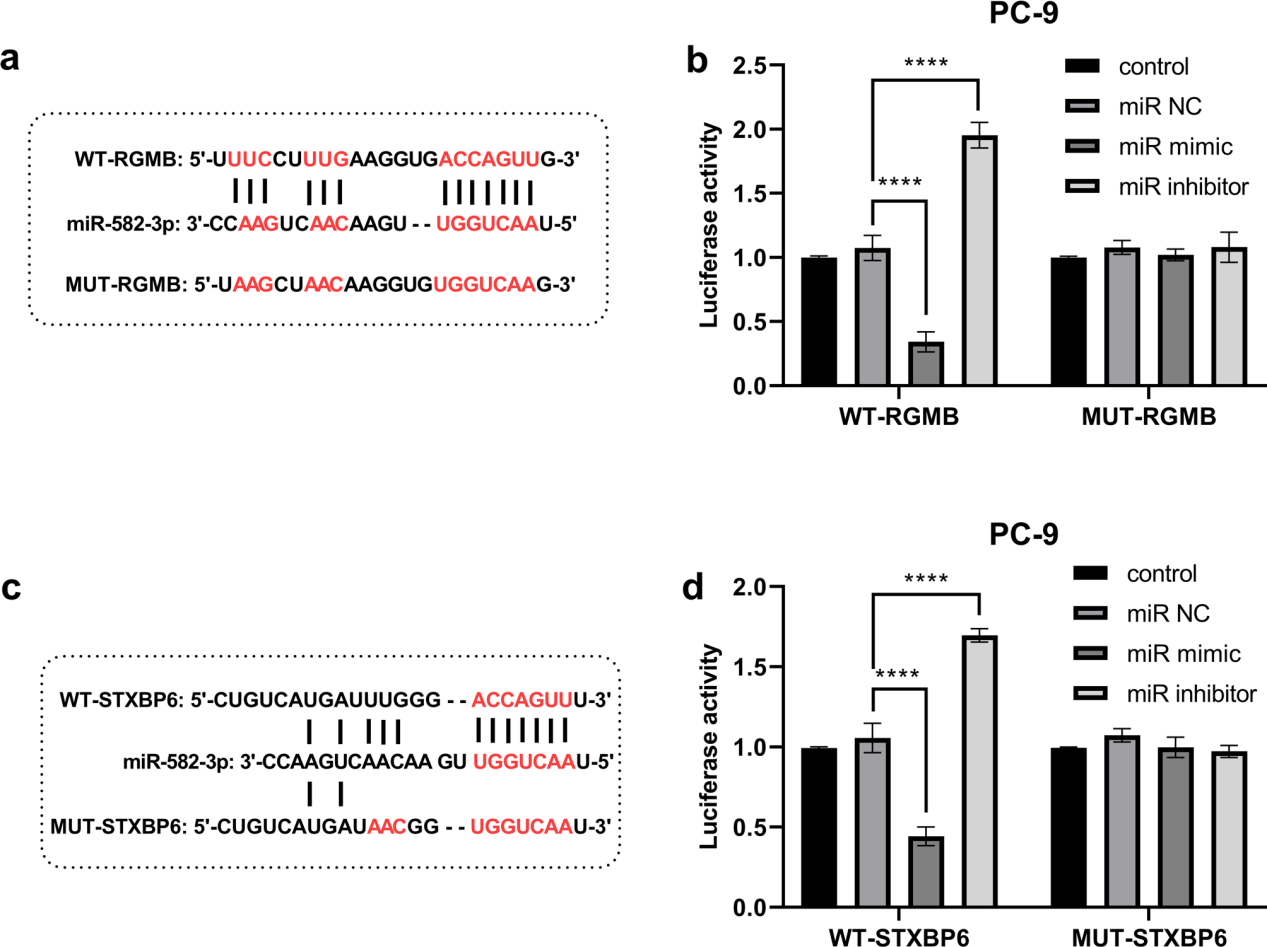
Validation of the targeting relationship of miR-582-3p with downstream target genes RGMB and STXBP6 in PC-9 lung cancer cells. (**a**) Complementary sequence of miR-582-3p with RGMB and MUT-RGMB sequence information. (**b**) Dual-luciferase reporter assay to validate the targeting relationship of miR-582-3p with RGMB. (**c**) STXBP6 with miR- 582-3p binding sequence information and MUT-STXBP6 sequence construction. (**d**) Dual luciferase reporter assay to validate the targeting relationship between miR-582-3p and STXBP6

The analysis of the proliferation of the two types of LUAD cells following transfection revealed a pronounced inhibition of abnormal proliferation upon overexpression of PRKCQ-AS1 in both cases (*P < 0.0001*). Furthermore, the abnormal proliferation of LUAD cells was significantly enhanced with the concurrent application of oe + miR-mimic (*P < 0.0001*, Fig. 4a and b). In addition, we observed that the expression of oe-PRKCQ-AS1 persistently inhibited the migratory and invasive capabilities of LUAD cells (*P < 0.0001*). Notably, the previously inhibited proliferation of LUAD cells was markedly restored following the simultaneous application of miR-mimic (*P < 0.0001*, Fig. 4c-d). It is inferred that PRKCQ-AS1 may regulate the progression of LUAD by antagonizing the action of miR-582-3p. According to the TargetScan database prediction, RGNB (Fig. 5a), STXBP6 (Fig. 5c) might all be downstream target genes of miR-582-3p. Dual luciferase reporter assays demonstrated that miR-582-3p was targeted to both RGMB (Fig. 5b, *P* *< 0.0001*) and STXBP6 (Fig. 5d, *P* *< 0.0001*), respectively.

## Discussion

Lung cancer ranks prominently among the most vulnerable cancers worldwide, with LUAD accounting for over 80% of these cases [22]. Despite the availability of various treatment modalities, LUAD is characterized by a dismal prognosis and rapid progression [23, 24]. Numerous studies have also shown that many patients diagnosed with LUAD experience prolonged recovery times and significant survival challenges, particularly in the late stage of treatment [25, 26]. Consequently, it is crucial to identify biomarkers associated with the progression and prognosis of LUAD, to explore the causes of poor prognosis of LUAD in order to understand the underlying causes of its poor outcomes and to address the biological challenges linked to its rapid advancement. Based on the results of an existing study [27], we collected and analyzed the expression in cancer tissues from LUAD patients. Surprisingly, we found that PRKCQ-AS1 was prominently downregulated in LUAD tissues, while miR-582-3p expression was noticeably increased. Statistics on the prognosis and 5-year survival of LUAD patients revealed that the average number of deaths was lower in the group with high expression of PRKCQ-AS1 versus low expression of miR-582-3p. Cox results showed that PRKCQ-AS1, miR-582-3p were independent factors affecting LUAD progression [28]. So far, we found preliminary evidence that PRKCQ-AS1, miR-582-3p may be involved in LUAD progression.

LncRNAs play a vital role in various metabolic processes and cellular functions within organisms [29, 30], particularly in the context of cancer progression [31, 32]. For example, PRKCQ-AS1 has been shown to engage with specific miRNAs to influence the recurrence of lymphoma [33] and modulate drug resistance in breast cancer by inducing autophagy through binding miR-361-5p [34]. Results from luciferase reporter assays indicate a similar interaction between PRKCQ-AS1 and miR-582-3p. Cellular functionality experiments showed that transfection with oe-PRKCQ-AS1 significantly mitigates the aberrant proliferative, migratory, and invasive tendencies of LUAD cells; however, simultaneous application of a miR-mimic resulted in an enhancement in proliferative capacity along with a restoration of the migratory and invasive capabilities in these cancer cells. This reciprocal effect has been occurred in various therapeutic contexts [35, 36], for example, overexpression of PRKCQ-AS1 has been found to impede the progression of thyroid cancer while simultaneously improving mitochondrial function [37]. Conversely, knockdown of PRKCQ-AS1 has been linked to abnormal proliferation and migration in colorectal cancer (CRC) cells [15]. In addition, miR-582-3p inhibition affects prostate cancer (PCa) cell function by silencing ORC6 expression [38], and overexpression of miR-582-3p accelerates smooth muscle cell proliferation and promotes carotid artery stenosis progression [39]. In addition, the study predicted target genes downstream of miR-582-3p that may be involved in the progression of LUAD by TargetScan database. Dual luciferase reporter assay demonstrated the presence of miR-582-3p in PC-9 lung adenocarcinoma cells in relation to STXBP6 and RGMB target binding. Other studies have also detected miR-582-3p targeting relationship with STXBP6 [40] or RGMB [18] in A549 cells as well. So far, we initially found the targeting relationship of PRKCQ-AS1/miR-582-3p regulatory axis and its target genes (STXBP6, RGMB) in lung adenocarcinoma cells. Therefore, it is necessary to study the molecular mechanisms of PRKCQ-AS1 and miR-582-3p for LUAD progression, and PRKCQ-AS1 or miR-582-3p is promising to be a therapeutic target and a basis for prognostic assessment to stop LUAD progression in the future.

However, it is regrettable that the study has several shortcomings. Although we tried our best to ensure the adequacy and representativeness of the study population, certain special populations (e.g., patients with severe underlying diseases who could not tolerate the relevant examinations) were difficult to be included in the study, which may affect the generalizability of the study results. We will consider including more populations with different characteristics for validation in the future. In addition, since PRKCQ-AS1 was lowly expressed in LUAD cells itself, and we observed a clear down-regulation trend. Therefore, we were only able to validate the regulatory relationships present in lung adenocarcinoma cells by overexpression and were unable to use loss-of-function experiments (si-PRKCQ-AS1). miR-582-3p is a lung cancer-associated regulator reported earlier [41], but we initially found that PRKCQ-AS1-targeted miR-582-3p was involved in the regulatory process of LUAD. However, we did not validate the molecular mechanism regarding PRKCQ-AS1 targeting miR-582-3p to regulate apoptosis and inflammatory response. Therefore, there is still a long way to go to explore the pathogenesis of lung adenocarcinoma. Our study found that overexpression of PRKCQ-AS1 inhibited LUAD cell proliferation, migration and invasion. Cell proliferation and other functions were restored after transfection with oe- PRKCQ-AS1 + miR-582-3p mimic. It has been found that miR-582-3p overexpression can reverse apoptosis induced by circRNA_PTPRA overexpression [19]. Therefore, it can be inferred that cancer cells may undergo increased apoptosis and delayed entry into the cell cycle when overexpressing PRKCQ-AS1. We will delve into the effects of PRKCQ-AS1 expression on apoptosis and cell cycle in lung adenocarcinoma cells in subsequent studies. In addition, it was found that miR-582-3p could also maintain lung cancer stem cell characteristics through Wnt/β-catenin signaling [42]. The results of osteoarthritis (OA) rat model found that miR-582-3p inhibited chondrocyte apoptosis and reduced pro-inflammatory cytokine production [43]. Thus, the topic of exploring the involvement of PRKCQ-AS1/miR-582-3p and its target genes in the lung adenocarcinoma cell cycle and inflammatory response [44] deserves further exploration. In addition, due to experimental design limitations, the study did not conduct an in-depth investigation into parallel pathways or other interactions involving PRKCQ-AS1 in LUAD regulation, which is regrettable.

In summary, it was found that down-regulated PRKCQ-AS1 and up-regulated miR-582-3p were present in LUAD patients and cells. PRKCQ-AS1 and miR-582-3p were independent factors affecting LUAD, and the expression of both was able to influence tumor differentiation, TNM staging and lymph node metastasis in LUAD patients. Overexpression of PRKCQ-AS1 in LUAD cells inhibited the aberrant proliferation, migration and invasion of cancer cells through down-regulation leading to miR-582-3p. Based on this inference, PRKCQ-AS1 is likely to be developed as a drug target for the prediction and treatment of LUAD. Yu et al. [45] reported that the predicted six copper-induced cell death (cuproposis)-associated lncRNAs might be used to predict the efficacy and prognosis of immunotherapy in LUAD patients. Studies have shown that the /IGF2BPs/PRMT7 axis, in which PRKCQ-AS1 is involved, is an ideal therapeutic target for the diagnosis and treatment of thyroid cancer [37]. PRKCQ-AS1 also promotes breast cancer cell apoptosis and impairs paclitaxel resistance through miR-361-5p/PIK3C3-mediated autophagy [34]. In addition, it has been shown that Erianin may exert antitumor effects in cisplatin-resistant LUAD cells by modulating the Wnt3 signaling pathway [46]. Therefore, in LUAD patients exhibiting PRKCQ-AS1 expression, targeting PRKCQ-AS1-promoting agents or overexpressing PRKCQ-AS1 mimics may mitigate malignant progression of the disease. However, clinical considerations such as drug off-target effects and safety must be duly evaluated. This study bridges the gap of PRKCQ-AS1/miR-582-3p regulatory axis in the study of LUAD and may provide a theoretical basis for future targeted therapy for LUAD.

## Electronic supplementary material

Below is the link to the electronic supplementary material.

Supplementary Material 1

## Data Availability

The datasets used and/or analysed during the current study are available from the corresponding author on reasonable request.
