# Supplementary material for: Clinical significance and biological function of PRKCQ-AS1/miR-582-3p expression in LUAD
Source: Hereditas. 2025 Jul 1;162:116. doi: 10.1186/s41065-025-00482-9 (PMC12211715; doi:10.1186/s41065-025-00482-9)
Supplement: Supplementary file 1 — Supplementary Material 1 [file 41065_2025_482_MOESM1_ESM.docx]

**
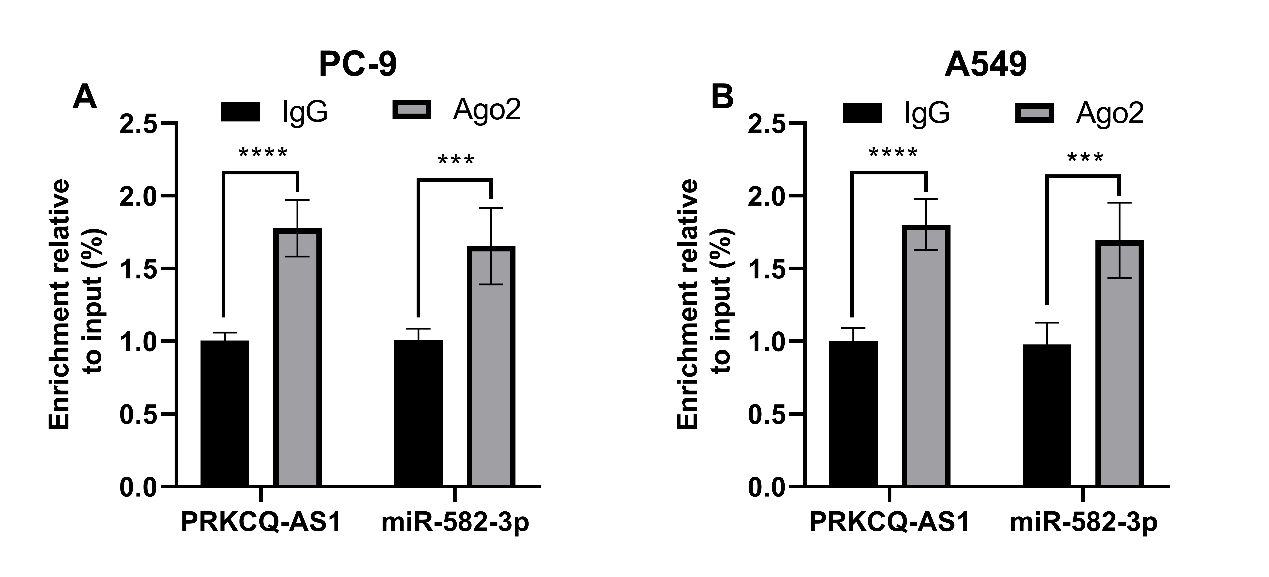
**

**Supplementary Figure 1** RNA immunoprecipitation validation of PRKCQ-AS1 interactions with its target gene miR-582-3p. a. Interaction of PRKCQ-AS1 with its target gene miR-582-3p presented in PC-9 cells. b. Validation of miR-582-3p targeting with PRKCQ-AS1 in A549 cells.
